# Offspring Production among the Extended Relatives of Samoan Men and *Fa'afafine*

**DOI:** 10.1371/journal.pone.0036088

**Published:** 2012-04-27

**Authors:** Doug P. VanderLaan, Deanna L. Forrester, Lanna J. Petterson, Paul L. Vasey

**Affiliations:** Department of Psychology, University of Lethbridge, Lethbridge, Alberta, Canada; Indiana University, United States of America

## Abstract

*Androphilia* refers to sexual attraction to adult males, whereas *gynephilia* refers to sexual attraction to adult females. Male androphilia is an evolutionary paradox. Its development is at least partially influenced by genetic factors, yet male androphiles exhibit lower reproductive output, thus raising the question of how genetic factors underlying its development persist. The sexual antagonism hypothesis posits that the fitness costs associated with genetic factors underlying male androphilia are offset because these same factors lead to elevated reproduction on the part of the female relatives of androphilic males. Western samples drawn from low fertility populations have yielded inconsistent results when testing this hypothesis. Some studies documented elevated reproduction among the matrilineal female kin of androphilic males, whereas others found such effects in the paternal line. Samoa is a high-fertility population in which individuals reproduce closer to their maximum capacities. This study compared the reproductive output of the paternal and maternal line grandmothers, aunts, and uncles of 86 Samoan androphilic males, known locally as *fa'afafine*, and 86 Samoan gynephilic males. Reproductive output was elevated in the paternal and maternal line grandmothers, but not aunts or uncles, of *fa'afafine*. These findings are consistent with the sexual antagonism hypothesis and suggest that male androphilia is associated with elevated reproduction among extended relatives in both the maternal and paternal line. Discussion focuses on how this study, in conjunction with the broader literature, informs various models for the evolution of male androphilia via elevated reproduction on the part of female kin.

## Introduction

The manner in which male same-sex sexual orientation is publicly expressed varies cross-culturally [1]. In Western societies, for example, men who are preferentially sexually attracted to other men typically identify as “gay” or “homosexual.” Such men exhibit what is referred to as an egalitarian pattern of same-sex sexual interaction that occurs between two males who are not markedly different in age or gender-related characteristics. As part of this egalitarian pattern, partners treat each other as social equals given that they both adopt culturally prescribed gender roles as men.

In contrast, male same-sex sexual orientation is publically expressed in a transgendered form in many non-Western societies. As the name suggests, same-sex sexually oriented males in such societies are typically transgendered in their appearance and mannerisms, and often occupy “alternative” gender role categories. These categories are distinguished linguistically from the gender-normative categories of “man” and “woman.” This transgendered pattern is associated with male same-sex sexual behavior that occurs between a male who is markedly gender-atypical (i.e., transgendered) and another who is more or less gender-typical for his own sex and adopts the culturally prescribed gender role of “man.” Thus, unlike the egalitarian pattern, partners exhibiting this transgendered pattern adopt different social roles and do not treat each other as social equals. Some contemporary examples include the *xanith* of Oman, the *hijra* of India, the *kathoey* of Thailand, the *travestí* of Brazil, the *fakafefine* of Tonga, and the *fa'afafine* of Samoa [1], [2].

Given these unique attributes, using terms that are associated with male same-sex sexual orientation in Western societies (e.g., gay, homosexual, or even men-who-have-sex-with-men) to describe the sexual orientation of transgendered males in these other societies would be misleading. It is, therefore, more accurate to use terminology that transcends culturally constructed concepts when examining hypotheses concerning the evolution of male same-sex sexual orientation within a cross-cultural framework, as we do here. Hence, to describe male same-sex sexual orientation, we use the term *androphilia*, which refers to sexual attraction and arousal toward adult males. To describe male opposite-sex sexual orientation, we use the term *gynephilia*, which refers to sexual attraction and arousal toward adult females.

Male individuals within numerous non-human primate species are known to exhibit same-sex sexual behavior (most commonly mounting) as part of a behavioral repertoire that includes opposite-sex sexual behavior as well [3], [4]. A small subset of human males appear to share this capacity for sexual arousal toward men and women, and engage in sexual behavior with members of both sexes [5]. For this subset of individuals, direct reproduction (i.e., passing on genes through offspring) is a possibility given that same-sex sexual behavior is accompanied by opposite-sex sexual behavior as well. As such, the non-human primate literature on same-sex sexual behavior might provide valuable insight into the evolution of human males' capacity to become aroused by, and engage in sexual interactions with, members of both sexes. The non-human primate literature is limited, however, with respect to its ability to inform evolutionary explanations for why a substantial minority of human males (less than, but up to, 5% cross-culturally) [6] exhibit life-long androphilia and exclusive to near-exclusive same-sex sexual behavior. This latter aspect of male same-sex sexual behavior is the evolutionary paradox that we focus on here.

This paradox is founded on three sets of empirical findings. First, studies of sexual orientation concordance rates among monozygotic and dizygotic twins indicate that the development of male androphilia is at least partially influenced by genetic factors [7]–[10]. Second, in Western societies, androphilic men exhibit lower reproductive output than gynephilic men [11], [12]. Meanwhile, transgendered androphilic males are unlikely to reproduce whatsoever [13], [14]. Third, prehistoric cave art and pottery suggest that male-male sexual activity is not evolutionarily recent [15]–[17]. Consequently, it is unclear how genetic factors underlying male androphilia persist from one generation to the next. The persistence of genetic factors for male androphilia over evolutionary time is a paradox in need of explanation given that natural selection is a process that favors the evolution of traits that facilitate reproductive success.

The sexual antagonism hypothesis is one potential resolution to this paradox [18]. Sexually antagonistic selection pertains to situations in which genetic factors that produce fitness costs when present in one sex result in fitness benefits when present in the other sex. In the present case, genetic factors for male androphilia might result in fitness costs when expressed in males, but conversely, result in fitness benefits in the form of elevated reproduction when expressed in females. In essence, the fitness benefits of increased reproduction on the part of female relatives of androphilic males would balance out the fitness costs of androphilic males' lack of reproduction, thus facilitating the persistence of genetic factors for male androphilia. Thus, the sexual antagonism hypothesis predicts that the female relatives of androphilic males should tend to produce more offspring than those of gynephilic males.

To date, several studies carried out in Western populations have compared the reproductive output of the extended relatives of male androphiles versus gynephiles. In two Italian samples, elevated reproduction was documented among the matrilineal female kin of androphilic men (i.e., mothers and maternal line grandmothers and aunts) [18], [19]. Likewise, a similar matrilineal effect was found in one British sample in which elevated reproduction was documented among the maternal aunts of androphilic men [20]. However, such matrilineal effects have not been replicated in other samples. In a separate British sample, androphilic males had significantly more aunts and uncles as well as cousins in the paternal, but not maternal, line [11]. Similarly, in a study from the USA comparing the reproductive output of maternal and paternal kin of androphilic and gynephilic males, elevated reproduction was documented among paternal grandmothers, but not the matrilineal female kin, of androphilic males [12].

One important limitation of this literature is its focus on samples drawn from Western populations. Such populations exhibit relatively low fertility [21]. In relation to this pattern of low fertility, individuals often exhibit “stopping rules” with respect to their reproductive behavior (e.g., cessation of reproduction once a certain number of children are produced or once at least one child of each sex is produced). Consequently, samples from low fertility populations can, in certain instances, produce anomalous patterns by obscuring the presence of biodemographic correlates of male sexual orientation [22], [23]. Discrepancies between Western studies of the familial patterning of male androphilia may, therefore, result from examining samples from low fertility populations. The susceptibility of these populations to producing anomalous familial patterning raises the possibility that some subset, or possibly all, of the aforementioned Western studies on male sexual orientation and family size are inaccurate (i.e., they do not provide clear indications of the reproductive output tendencies of androphilic males' extended relatives). Hence, examining the reproductive output of androphilic and gynephilic males' kin in a high fertility population in which individuals are more likely to be reproducing closer to their maximum capacities could provide valuable insight.

The Samoan population is suitable for such an examination. The Samoan population is characterized by higher fertility than the West [21]. Furthermore, male androphilia shows developmental commonalities in the West and Samoa. As in the West, male androphilia in Samoa is associated with elevated recall of childhood gender-atypical behavior [24], [25] and traits of separation anxiety [26], [27], later birth order [28]–[30], greater numbers of older biological brothers [29], [31], and greater numbers of siblings [18], [29], [30].

In Samoa, androphilic males are known locally as *fa'afafine*. Translated literally, *fa'afafine* means “in the manner of a woman.” Status as *fa'afafine* is initially assigned on the basis of gender-atypical behavior beginning in childhood [32]–[35]. In adulthood, *fa'afafine* are extremely feminine in their appearance and mannerisms [25], [34], [36], [37]. Effeminate patterns of behavior, not adult sexual orientation, are the primary basis for having *fa'afafine* status (as opposed to status as “man” or “woman”). Nevertheless, *fa'afafine* are overwhelmingly androphilic in adulthood and only engage in sexual behavior with masculine males who identify as “straight men” (i.e., *fa'afafine* do not engage in sexual behavior with one another); exceptions to these rules are exceedingly rare to the point where they are considered questionable and highly suspect by Samoans both within and outside of the *fa'afafine* community [14], [37]. Also, it is important to note that the vast majority of *fa'afafine* are not transsexual because they do not experience dysphoria with respect to their genitalia [35].

In a Samoan cultural context, “straight men” are those who self-identify as men and are masculine with respect to gender role presentation. Inclusion in this category is not contingent on exclusive sexual activity with women. Most self-identified straight men are gynephilic, but may engage in sexual activity with *fa'afafine* or even other straight men on a temporary basis, particularly if female sexual partners are unavailable. It has been noted, and is probably underappreciated by many researchers, that one of the most cross-culturally variable aspects of gynephilic male sexuality appears to be their willingness to engage in sexual activity with their less preferred sex, namely, other males [6]. Indeed, our participants informed us that many straight men in Samoa have engaged in sexual interactions with *fa'afafine* at least once in their lives [also see 37]. While this seems paradoxical to many Western observers, it is important to keep in mind that *fa'afafine* represent much closer facsimiles of women—the preferred sex of gynephilic men—than do Western gay men. As such, when constructing a participant group of gynephilic Samoan men to compare to androphilic *fa'afafine*, it is appropriate to use sexual attraction as a basis for inclusion, not sexual behavior. This measure provides a window on sexual orientation in the absence of real-world constraints. In contrast, using sexual behavior as a measure of sexual orientation is confounded by the participant's ability, or lack thereof, to access female sexual partners. Self-identification as a straight man is also an inadequate basis for forming a comparison group given that although the majority of such men are gynephilic, a small minority may be more or less equally attracted to both sexes or exhibit a preference for males.

Previous research has shown repeatedly that the mothers of *fa'afafine* have significantly higher reproductive output than those of gynephilic men [29], [30]. The current study compared the reproductive output of the maternal and paternal line male and female extended relatives (i.e., grandmothers, aunts, and uncles) of Samoan *fa'afafine* and gynephilic males. It did so to shed light on whether male androphilia in this relatively high fertility population is associated with elevated reproduction in the maternal line, paternal line, or both.

## Methods

### Ethics Statement

This research was approved by the University of Lethbridge Human Subjects Research Ethics Committee. Informed written consent was obtained from all participants.

### Participants and Measures

Data were collected on Samoa's most populous islands, Upolu and Savai'i, during July–September, 2008. Participants were recruited through a network sampling procedure, which involved contacting initial participants, then obtaining referrals from them to additional participants who, in turn, provided further referrals, and so on. The rate of participation for all groups was greater than 90%. All participants were interviewed in English or Samoan, depending on their preference, using a standardized questionnaire. The questionnaire included questions concerning gender identity (i.e., status as a man or *fa'afafine*), age, sexual orientation, and numbers of children produced by various categories of kin (i.e., maternal and paternal grandmothers, aunts, and uncles).

Participants included 86 gynephilic males (*M* ± *SD* age: 29.80±9.61) and 86 *fa'afafine* (*M* ± *SD* age: 29.60±8.44). Across the entire sample, none of the participants were brothers or first cousins. Groups were comparable with respect to age (*t*[170] = .15, *p* = .88). Kinsey ratings of sexual feelings toward males (i.e., men and/or *fa'afafine*) and females (i.e., women) during the previous year were obtained. Specifically, participants were asked the following question: “Which statement best describes your sexual feelings during the last year?” Participants then selected one of the following seven possible responses: “sexual feelings only toward females” (Kinsey rating = 0), “most sexual feelings toward females, but an occasional fantasy about males” (Kinsey rating = 1), “most sexual feelings toward females, but some definite fantasy about males” (Kinsey rating = 2), “sexual feelings about equally divided between males and females with no strong preference for one or the other” (Kinsey rating = 3), “most sexual feelings toward males, but some definite fantasy about females” (Kinsey rating = 4), “most sexual feelings toward males, but an occasional fantasy about females” (Kinsey rating = 5), or “sexual feelings only toward males” (Kinsey rating = 6). Samoans, both inside and outside the *fa'afafine* community, recognize that *fa'afafine* are biological males that are socially distinct from men and women. Nevertheless, for the sake of consistency, participants were told, prior to answering questions pertaining to the Kinsey ratings, that the category “males” included straight men and/or *fa'afafine*, whereas the category “females” included women. All 86 gynephilic males described their sexual feelings as exclusively gynephilic (Kinsey rating = 0). Of the *fa'afafine*, 84 (97.7%) described their sexual feelings as exclusively androphilic (Kinsey rating = 6), and two (2.3%) reported most sexual feelings toward males, but an occasional fantasy about females (Kinsey rating = 5).

Finally, following previous studies [18]–[20], participants were asked to report the number of children born to their grandmothers and each of their aunts and uncles (i.e., not including adopted or step-family) for the maternal and paternal sides of their families. From this information, for each participant, we calculated the mean number of children produced by their maternal aunts, maternal uncles, paternal aunts, and paternal uncles. Importantly, Samoans often emigrate to countries with lower fertility populations (e.g., Australia, New Zealand, USA) for the entirety, or a portion, of their reproductive lives. There is reason to suspect that such emigration lowers fertility. Although the US territory of American Samoa is populated principally by ethnic Samoans, its fertility rate is lower than in the politically autonomous portion of the Samoan archipelago where we conducted the present study [21]. Consequently, our analyses focused on the reproduction of grandmothers, aunts, and uncles for whom all offspring were born in Samoa.

## Results

The offspring production of paternal and maternal line grandmothers, aunts, and uncles in Samoan androphilic (i.e., *fa'afafine*) versus gynephilic male probands was compared using independent *t*-tests. Comparisons were made using SPSS, version 19. An alpha level of 0.008 was used for determining statistical significance in order to maintain a Type I Error rate of 0.05 across the six comparisons. These comparisons are summarized in Table 1, and showed that the paternal and maternal grandmothers, but not aunts or uncles, of androphilic males exhibited elevated reproduction.

**Table 1:** Sample sizes (n), means (M), standard deviations (SD), t-values (t), degrees of freedom (df), p-values (p), and effect sizes (Cohen's d) pertaining to comparisons of numbers of offspring produced by the paternal and maternal line grandmothers, aunts, and uncles of Samoan fa'afafine versus gynephilic males.

	*Fa'afafine*			Gynephilic males						
	*n*	*M*	*SD*	*n*	*M*	*SD*	*t*	*df*	*p* c	Cohen's *d*
Paternal grandmothers	85	6.35	2.46	83	4.99	1.71	4.19	150^a^	<0.001	0.64
Paternal aunts	66	4.93	3.42	73	4.63	3.80	0.49	137	0.625	0.08
Paternal uncles	70	4.84	3.24	74	4.70	2.90	0.27	142	0.791	0.05
Maternal grandmothers	86	7.29	2.97	86	5.47	2.08	4.67	152.3^b^	<0.001	0.71
Maternal aunts	65	5.28	3.70	76	4.92	3.43	0.61	139	0.546	0.10
Maternal uncles	74	5.31	5.01	79	5.78	4.12	−0.64	151	0.522	−0.10

a Degrees of freedom adjusted based on Levene's test for equality of variances: *F* = 10.80, *p* = .001.

b Degrees of freedom adjusted based on Levene's test for equality of variances: *F* = 6.09, *p* = .015.

c Two-tailed *p*-value.

## Discussion

Some studies conducted in low fertility, Western populations reported elevated offspring production among the matrilineal female kin of androphilic males [18]–[20] while others reported elevated offspring production among female paternal relatives [11], [12]. The present study compared the number of children born to the paternal and maternal line grandmothers, aunts, and uncles of androphilic (i.e., *fa'afafine*) versus gynephilic males in Samoa, a relatively high fertility population in which individuals are more likely to reproduce closer to their maximum capacities. These comparisons indicated that offspring production in Samoa is elevated among the maternal and paternal line grandmothers, but not aunts and uncles, of androphilic males.

One may wonder whether the lack of group differences for aunts and uncles is due to the possibility that these relative categories are less likely to have completed their reproductive careers compared to grandmothers. The samples presented here were age-matched. As such, if the reproduction of androphilic males' relatives was elevated throughout their reproductive careers, then group differences should have emerged. The only manner in which incompleteness of reproductive careers can account for the lack of group differences for aunts and uncles is, therefore, if the kin of *fa'afafine* have greater reproductive output than the kin of gynephilic males toward the latter part of their reproductive careers. Future research may benefit from focusing on the reproductive output of the extended relatives of androphilic versus gynephilic males as a function of relatives' ages.

One may also wonder whether elevated reproduction by the maternal and paternal grandmothers of *fa'afafine* supports the sexual antagonism hypothesis given that the reproduction of grandmothers is naturally confounded with that of grandfathers. As such, it is difficult to discern from this study alone as to whether elevated reproduction is strictly limited to the female relatives of androphilic males. That said, in the present study and all previous studies comparing the offspring production of the extended relatives (i.e., grandmothers, aunts, and uncles) of androphilic versus gynephilic males, the only categories of androphilic male relatives to show elevated reproduction were those comprised partially (i.e., reproduction of aunts and uncles combined) [11] or entirely of female kin [12], [18]–[20]. In addition, the mothers of androphilic males appear to have greater numbers of children compared to the mothers of gynephilic males in the West [18], [19] as well as in Samoa [29], [30]. Based on this information, the sexual antagonism hypothesis is still a tenable explanation for the evolution of male androphilia.

The main strength of the study presented here was its consideration of reproductive output among the relatives of androphilic and gynephilic males within a population that has higher fertility compared to the West. As mentioned previously, studies conducted in the West had reported elevated extended family size effects in either the paternal or maternal line of androphilic males, but not both. These discrepancies possibly exist because the use of “stopping rules” that curtail reproduction make low fertility populations susceptible to producing anomalous patterns with respect to biodemographic correlates of male sexual orientation. Anomalous patterns would be less likely to occur in the Samoan population because it exhibits relatively higher fertility and, as such, individuals are less likely employ “stopping rules” that curtail offspring production early in their reproductive careers. The data presented here showed that male androphilia in Samoa is associated with elevated reproduction in both maternal and paternal grandmothers. Hence, if the Samoan population is relatively free of susceptibility to anomalous patterns, then the present study indicates that male androphilia is actually associated with larger extended family size in both the maternal and paternal line. Replications of this research in various populations would further help to identify factors that influence inter-population differences in the expression of elevated reproduction among the relatives of androphilic males, and to discern which categories of kin show elevated reproduction most reliably.

Identifying that elevated female reproduction is most likely inherent to both the maternal and paternal lines of androphilic males has important implications regarding the proximate bases of this pattern. The sexual antagonism hypothesis suggests that the proximate basis of this elevated reproduction is genetic. Previous debate in the literature concerning the genetic basis of such sexually antagonistic genetic factors has centered around the issue of whether these factors are located on the X chromosome. Such X-linkage was suggested based on a number of findings. First, as mentioned previously, some studies reported that elevated reproduction on the part of androphilic males' relatives was specific to the matrilineal female kin, and noted that this pattern would depend on X-linkage because males share this chromosome with their matrilineal kin only [18]–[20]. Second, this suggestion is in agreement with other studies indicating that genetic factors on the X chromosome are associated with the etiology of male androphilia. For example, in several Western samples, androphilic male probands show preponderances of androphilic male relatives (i.e., uncles and cousins) in the maternal, but not paternal, line [18], [20], [38], [39], a pattern that would depend on X-linkage. Moreover, two genetic studies have documented differences in the X chromosomes of androphilic and gynephilic males at the Xq28 locus [38], [39], while another study has indicated that activation (i.e., epigenetic) processes related to genetic factors on the X chromosome are important [40].

At the same time, however, findings from various studies, including the present study, raise doubt about the existence of sexually antagonistic, X-linked genetic factors in the development and evolution of male androphilia. To begin with, androphilic male probands have shown preponderances of androphilic male relatives in both the maternal and paternal lines in some samples [12], [41]. Also, two genetic studies did not show X-chromosome differences between androphilic and gynephilic males [42], [43]. The original findings of male sexual orientation differences at Xq28 may reflect Type I Error due to the fact that genotyping was performed using microsatellite markers, which have high error rates [44]. Furthermore, linkage disequilibrium (i.e., non-random allelic association) between the markers was not assessed and taken into account during analysis, which can also result in false positives [45]. Lastly, and perhaps most importantly, elevated reproduction does not appear to be limited to the matrilineal female kin of androphilic males. As the present study and other studies [11], [12] have shown, elevated reproduction exists among the patrilineal female kin of androphilic males as well. Based on these findings, one would argue that male androphilia is not primarily an X-linked phenomenon, and that X-linked sexual antagonism might not be the form of selection responsible for its evolution. One might instead argue that sexually antagonistic genetic factors are present on the autosomal chromosomes because androphilic males share genetic factors on these chromosomes with both paternal and maternal relatives. Indeed, autosomal linkage of sexually antagonistic genetic factors favoring the evolution of male androphilia is plausible given previously reported mathematical models of sexually antagonistic selection for the evolution of male androphilia [46].

It is possible that genetic factors underlying male androphilia do not have any influence on female reproduction. Rather, the female relatives of male androphiles may simply have a propensity for elevated reproduction due to social mechanisms that are not under genetic influence. Specifically, if certain families have social norms that encourage offspring production and larger family sizes, then elevated reproduction among the female, but not male, members of such families is likely. This is because male reproductive output is limited by access to females who are willing to reproduce [47]. As such, the reproductive outputs of the gynephilic male relatives of androphilic males would be relatively more constrained because their female sexual partners might come from families that do not have social norms that encourage offspring production and larger family size. If this line of reasoning were correct, it would explain why the current study and all previous studies have repeatedly found elevated reproduction among the female, but not male, relatives of androphilic males. As a result of elevated female reproduction, shared genetic factors associated wtih male androphilia would gain fitness benefits within families with social norms encouraging reproduction. These fitness benefits would accrue without the genetic factors associated with male androphilia exerting any shared influence over reproduction in the female kin of androphilic males. To discern whether this scenario accounts for why androphilic males tend to belong to families with elevated female reproduction, future research should examine whether such elevated reproduction is owing to family social norms related to reproductive output.

It is also noteworthy that a number of studies have demonstrated that Samoan *fa'afafine* are more willing to help care for their nieces and nephews than Samoan women and gynephilic males [14], [36], [48]–[50]. These avuncular (uncle-like) tendencies are expressed by *fa'afafine* in an economical, efficient, reliable, and precise manner, all of which are indicative of past selection for adaptive design [48], [51]. It is possible that such elevated avuncularity on the part of *fa'afafine* contributes to the reproduction of kin and that genetic factors underlying male androphilia accrue fitness in this manner as well. As such, genetic factors associated with an adaptive avuncular androphilic male phenotype may influence reproductive output in the female kin of male androphiles, but this relationship may be mediated by the phenotypic expression of elevated altruism toward nieces and nephews by androphilic males. Interestingly, one study focusing on monetary donations found that *fa'afafine* allocate more money toward younger siblings' daughters in particular [50]. A bias toward investing in nieces on the part of *fa'afafine* could be the most adaptive means of maximizing fitness given that the female, but not male, kin of androphilic males seem to exhibit elevated reproduction. Future research should, therefore, examine whether the kin investment tendencies of androphilic males promote the reproduction of female kin in particular.

The increased interest in investing in kin exhibited by *fa'afafine* might also be related to increased knowledge about kin. If so, then the androphilic *fa'afafine* might be better informed about their extended family members' reproductive outputs, thus confounding comparisons of androphilic and gynephilic males' relatives' reproduction. For example, greater knowledge of relatives' reproductive outputs could lead to more reliable reporting of offspring who died at a young age. Such confounds could contribute toward the impression of greater fertility among the relatives of androphilic males. Future research may limit such confounds by corroborating probands' reports of their relatives' reproductive outputs with those of knowledgeable relatives (e.g., mothers, fathers, siblings). Furthermore, such reports could be expanded to include additional pertinent information regarding relatives' offspring. For example, number of live births is a proxy for reproductive success, however, offspring survival (and subsequent reproduction) are critical aspects of relatives' reproductive success that influence the biological fitness and evolution of genetic factors underlying male androphilia. Consequently, in addition to collecting information about live births, it would be worthwhile if future studies also obtained information concerning offspring survival.

## References

[1] Murray SO (2000). Homosexualities.

[2] Herdt G (1996). Third sex, third gender: Beyond sexual dimorphism in culture and history.

[3] Dixson A, Polani A (2010). Homosexual behaviour in primates.. Animal homosexuality: A biosocial perspective.

[4] Vasey PL (1995). Homosexual behaviour in primates: A review of evidence and theory.. Int J Primatol.

[5] Rosenthal AM, Sylva D, Safron A, Bailey JM (2012). The male bisexuality debate revisited: Some bisexual men have bisexual arousal patterns.. Arch Sex Behav.

[6] Whitam FL (1983). Culturally invariable properties of male homosexuality: Tentative conclusions from cross-cultural research.. Arch Sex Behav.

[7] Alanko K, Santtila P, Harlaar N, Witting K, Varjoen M (2010). Common genetic effects of gender atypical behavior in childhood and sexual orientation in adulthood: A study of Finnish twins.. Arch Sex Behav.

[8] Bailey JM, Dunne MP, Martin NG (2000). Genetics and environmental influences on sexual orientation and its correlates in an Australian twin sample.. J Pers Soc Psychol.

[9] Kendler KS, Thornton LM, Gilman SE, Kessler RC (2000). Sexual orientation in a US national sample of twin and non-twin sibling pairs.. Am J Psychiatry.

[10] Långström N, Rahman Q, Carlström E, Lichtenstein P (2010). Genetic and environmental effects on same-sex sexual behavior: A population study of twins in Sweden.. Arch Sex Behav.

[11] King MD, Green J, Osborn DPJ, Arkell J, Hetherton J (2005). Family size in white gay and heterosexual men.. Arch Sex Behav.

[12] Schwartz G, Kim RM, Kolundziji AB, Rieger G, Sanders AR (2010). Biodemographic and physical correlates of sexual orientation in men.. Arch Sex Behav.

[13] LeVay S (1996). Queer science: The use and abuse of research into homosexuality.

[14] Vasey PL, VanderLaan DP (2010). Avuncular tendencies and the evolution of male androphilia in Samoan *fa'afafine*.. Arch Sex Behav.

[15] Larco Hoyle R (1998). Arte erotico en el antiguo Peru.

[16] Nash G, Bevan L (2001). The subversive male: Homosexual and bestial images on European mesolithic rock art.. Indecent exposure: Sexuality, society and the archaeological record.

[17] Yates T, Tilley C (1993). Frameworks for an archaeology of the body.. Interpretive archaeology.

[18] Camperio-Ciani A, Corna F, Capiluppi C (2004). Evidence for maternally inherited factors favoring male homosexuality and promoting female fecundity.. Proc R Soc B.

[19] Iemmola F, Camperio Ciani A (2009). New evidence of genetic factors influencing sexual orientation in men: Female fecundity increase in the maternal line.. Arch Sex Behav.

[20] Rahman Q, Collins A, Morrison M, Orrells JC, Cadinouche K (2008). Maternal inheritance and familial fecundity factors in male homosexuality.. Arch Sex Behav.

[21] Central Intelligence Agency (2011). The World Factbook.. https://www.cia.gov/library/publications/the-world-factbook/rankorder/2127rank.html.

[22] Blanchard R, Lippa RA (2007). Birth order, sibling sex ratio, handedness, and sexual orientation of male and female participants in a BBC Internet research project.. Arch Sex Behav.

[23] Zucker KJ, Blanchard R, Kim T-S, Pae C-U, Lee C (2007). Birth order and sibling sex ratio in homosexual transsexual South Korean males: Effects of the male-preference stopping rule.. Psychiatry Clin Neurosci.

[24] Bailey JM, Zucker KJ (1995). Childhood sex-typed behavior and sexual orientation: A conceptual analysis and quantitative review.. Dev Psychol.

[25] Bartlett NH, Vasey PL (2006). A retrospective study of childhood gender-atypical behavior in Samoan *fa'afafine*.. Arch Sex Behav.

[26] VanderLaan DP, Gothreau LM, Bartlett NH, Vasey PL (2011). Recalled separation anxiety and gender atypicality in childhood: A study of Canadian heterosexual and homosexual men and women.. Arch Sex Behav.

[27] Vasey PL, VanderLaan DP, Gothreau LM, Bartlett NH (2011). Traits of separation anxiety in childhood: A retrospective study of Samoan men, women, and *fa'afafine*.. Arch Sex Behav.

[28] Blanchard R (2004). Quantitative and theoretical analyses of the relation between older brothers and homosexuality in men.. J Theor Biol.

[29] VanderLaan DP, Vasey PL (2011). Male sexual orientation in Independent Samoa: Evidence for fraternal birth order and maternal fecundity effects.. Arch Sex Behav.

[30] Vasey PL, VanderLaan DP (2007). Birth order and male androphilia in Samoan *fa'afafine*.. Proc R Soc Lond B Biol Sci.

[31] Bogaert AF, Skorska M (2011). Sexual orientation, fraternal birth order, and the maternal immune hypothesis: A review.. Front Neuroendrocrinol.

[32] Poasa K (1992). The Samoan *fa'afafine*: One case study and a discussion of transsexualism.. J Psychol Human Sex.

[33] Schmidt J (2003). Paradise lost? Social change and *fa'afafine* in Samoa.. Curr Sociol.

[34] Shore B, Ortner SB, Whitehead H (1981). Sexuality and gender in Samoa: Conceptions and missed conceptions in sexual meaning.. The Cultural Construction of Gender and Sexuality.

[35] Vasey PL, Bartlett NH (2007). What can the Samoan *fa'afafine* teach us about the Western concept of “Gender Identity Disorder in Childhood”?. Perspect Biol Med.

[36] Vasey PL, Pocock DS, VanderLaan DP (2007). Kin selection and male androphilia in Samoan *fa'afafine*.. Evol Hum Behav.

[37] Croall H, Wunderman E (Directors) (1999). Paradise bent: Gender diversity in Samoa [Motion picture].

[38] Hamer DH, Hu S, Magnunson VL, Hu N, Pattattucci AM (1993). A linkage between DNA markers on the X-chromosome and male sexual orientation.. Science.

[39] Hu S, Pattatuci A, Patterson C, Li L, Fulker DW (1995). Linkage between sexual orientation and chromosome Xq28 in males but not in females.. Nat Genet.

[40] Bocklandt S, Horvath S, Vilain E, Hamer DH (2006). Extreme skewing of X chromosome inactivation in mothers of homosexual men.. Hum Genet.

[41] Bailey JM, Pillard RC, Dawood K, Miller MB, Farrer LA (1999). A family history study of male sexual orientation using three independent samples.. Behav Genet.

[42] Mustanski BS, Dupree MG, Nievergelt CM, Bocklandt S, Schork NJ (2005). A genomewide scan of male sexual orientation.. Hum Genet.

[43] Rice G, Anderson C, Risch N, Ebers G (1999). Male homosexuality: Absence of linkage to microsatellite markers at Xq28.. Science.

[44] Weeks DE, Conley YP, Ferrell RE, Mah TS, Gorin MB (2002). A tale of two genotypes: Consistency between two high-throughput genotyping centers.. Genome Res.

[45] Huang Q, Shete S, Amos CI (2004). Ignoring linkage disequilibrium among tightly linked markers induces false-positive evidence of linkage for affected sib pair analysis.. Am J Hum Genet.

[46] Gavrilets S, Rice WR (2006). Genetic models of homosexuality: Generating testable predictions.. Proc R Soc B.

[47] Trivers RL, Campbell B (1972). Parental investment and sexual selection.. Sexual selection and the descent of man 1871–1971.

[48] VanderLaan DP, Vasey PL (in press). Relationship status and elevated avuncularity in Samoan *fa'afafine*.. Pers Rel.

[49] Vasey PL, VanderLaan DP (2009). Materteral and avuncular tendencies in Samoa: A comparative study of women, men, and *fa'afafine*.. Hum Nat.

[50] Vasey PL, VanderLaan DP (2010). Monetary exchanges with nieces and nephews: A comparison of Samoan men, women, and *fa'afafine*.. Evol Hum Behav.

[51] Vasey PL, VanderLaan DP (2010). An adaptive cognitive dissociation between willingness to help kin and non-kin in Samoan *fa'afafine*.. Psych Sci.

